# Robust imputation method for missing values in microarray data

**DOI:** 10.1186/1471-2105-8-S2-S6

**Published:** 2007-05-03

**Authors:** Dankyu Yoon, Eun-Kyung Lee, Taesung Park

**Affiliations:** 1 Interdisciplinary Program in Bioinformatics, Seoul National University, Seoul, Korea; 2 Department of Statistics, College of Natural Science, Seoul National University, San 56-1, Shin Lim-Dong, Kwanak-ku, Seoul, 151-742, Korea

## Abstract

**Background:**

When analyzing microarray gene expression data, missing values are often encountered. Most multivariate statistical methods proposed for microarray data analysis cannot be applied when the data have missing values. Numerous imputation algorithms have been proposed to estimate the missing values. In this study, we develop a robust least squares estimation with principal components (RLSP) method by extending the local least square imputation (LLSimpute) method. The basic idea of our method is to employ quantile regression to estimate the missing values, using the estimated principal components of a selected set of similar genes.

**Results:**

Using the normalized root mean squares error, the performance of the proposed method was evaluated and compared with other previously proposed imputation methods. The proposed RLSP method clearly outperformed the weighted *k*-nearest neighbors imputation (kNNimpute) method and LLSimpute method, and showed competitive results with Bayesian principal component analysis (BPCA) method.

**Conclusion:**

Adapting the principal components of the selected genes and employing the quantile regression model improved the robustness and accuracy of missing value imputation. Thus, the proposed RLSP method is, according to our empirical studies, more robust and accurate than the widely used kNNimpute and LLSimpute methods.

## Background

Microarray experiment technique has been successfully applied to a variety of biological studies including cancer classification, discovery of the unknown gene function, and identification of effects of a specific therapy. When analyzing microarray data, we often face missing values due to various factors such as scratches on the slide, spotting problems, dusts, experimental errors, and so on. In practice, every experiment contains missing entries and sometimes more than 90% of the genes in the microarray experiment are affected [1]. Moreover, most of the classic multivariate analysis methods for microarray data cannot be used when the data have missing values. Therefore, we need to treat missing values appropriately.

An easy way to handle missing data is to repeat the whole experiment. However, often it is not a realistic option secondary to economic limitations and/or scarcity of available biological material [2]. Accordingly, many missing value estimation methods have been developed. The weighted *k*-nearest neighbors imputation method (kNNimpute) selects genes with expression profiles similar to the gene of interest to impute missing values [3]. The singular value decomposition method (SVDimpute) employs a singular value decomposition to obtain to a set of mutually orthogonal patterns that can be linearly combined to approximate the expression of all genes in the data set [3]. In a comparative study presented by Troyanskaya et al. [3], kNNimpute is more robust and accurate than SVDimpute.

Least squares imputation (LSimpute) is a regression-based method using the correlation between both genes and arrays [2]. LSimpute showed best performance when data have a strong local correlation structure. Local least squares imputation (LLSimpute) is an extension of LSimpute method which selects *k *similar genes by *L*_2_-norm or Pearson correlation and applies multiple regression to impute missing values [4].

Bayesian principal component analysis (BPCA) uses a Bayesian estimation algorithm to predict missing values [5]. BPCA suggests using the number of samples minus 1 as the number of principal axes. Since BPCA uses an EM-like repetitive algorithm to estimate missing values, it needs intensive computations to impute missing values. Gaussian mixture imputation (GMCimpute) estimates missing values using Gaussian mixture and model averaging [1]. Collateral missing value imputation (CMVE) [6] predicts missing values based on a multiple covariance-based imputation matrices and performs imputation using least square regression and linear programming methods.

Recently, several imputation methods using a priori information to impute missing values have been proposed such as a set theoretic framework approach based on projection onto convex sets (POCS) [7] and an approach based on the functional similarities of gene ontology [8]. While most traditional missing imputation methods treated spots as binary value such as missing or present, weighted nearest neighbours method (WeNNI) adopted a continuous spot quality weight for the missing value estimation [9].

Among these methods, kNNimpute, LSimpute and LLSimpute are most commonly used because they are easy to apply with less computational burdens. Note that LSimpute and LLSimpute are regression based methods and kNNimpute can also be regarded as a regression based method for the simple intercept model. In this paper, we focus on these regression based methods and present their improvements.

kNNimpute and LLSimpute both use the *k *selected genes to estimate missing values. Kim *et al*. [4] showed LLSimpute performed well for a large value of *k*, say over 200. However, it is inefficient to use such a large number of genes to estimate one missing value from a practical point of view. Furthermore, there is no guarantee that the selected *k *is sufficiently large enough for LLSimpute to perform well. Surprisingly, the performance of LLSimpute becomes very poor when *k *is close to the number of samples.

On the other hand, kNNimpute performs well with relatively small values of *k*. For example, kNNimpute suggests using 10 or 15 similar genes. However, kNNimpute performs poorly when *k *is too small or too large. Its performance depends on the sample size and the correlations between genes. Therefore, kNNimpute is negatively affected by a badly chosen *k*.

To overcome the limitations of these regression based imputation methods, we propose the robust least square estimation with principal components (RLSP) method. RLSP is an improved version of LLSimpute. We use the estimated principal components of the selected genes and apply quantile regression to estimate missing values with the estimated principal components. Note that the most imputation methods are not robust to outliers. RLSP performs well even when *k *is small. Moreover RLSP shows similar performance with LLSimpute when *k *is large. Therefore, RLSP is more robust to the choice of *k*.

The normalized root mean squared error (NRMSE) is used to evaluate the differences in performances between the proposed RLSP method and the other imputation methods for various missing rates [4]. The RLSP method clearly outperforms the LLSimpute method.

## Methods

A whole gene expression profile is represented by a *G *× *N *matrix, **Y**, where the rows correspond to the genes, the columns correspond to the experiments (samples), and the entry *Y*_*i*,*j *_is the expression level of gene *i *in experiment *j*. For simplicity, we assume that the target gene vector g* has a missing value at the first sample, denoted by *α*. For the *k *selected similar genes, let gsj
 MathType@MTEF@5@5@+=feaafiart1ev1aaatCvAUfKttLearuWrP9MDH5MBPbIqV92AaeXatLxBI9gBaebbnrfifHhDYfgasaacH8akY=wiFfYdH8Gipec8Eeeu0xXdbba9frFj0=OqFfea0dXdd9vqai=hGuQ8kuc9pgc9s8qqaq=dirpe0xb9q8qiLsFr0=vr0=vr0dc8meaabaqaciaacaGaaeqabaqabeGadaaakeaacqWHNbWzdaWgaaWcbaGaem4Cam3aaSbaaWqaaiabdQgaQbqabaaaleqaaaaa@3137@ be a *N *× 1 vector consisting of the *j*th selected genes with its first element wsj
 MathType@MTEF@5@5@+=feaafiart1ev1aaatCvAUfKttLearuWrP9MDH5MBPbIqV92AaeXatLxBI9gBaebbnrfifHhDYfgasaacH8akY=wiFfYdH8Gipec8Eeeu0xXdbba9frFj0=OqFfea0dXdd9vqai=hGuQ8kuc9pgc9s8qqaq=dirpe0xb9q8qiLsFr0=vr0=vr0dc8meaabaqaciaacaGaaeqabaqabeGadaaakeaacqWG3bWDdaWgaaWcbaGaem4Cam3aaSbaaWqaaiabdQgaQbqabaaaleqaaaaa@3153@, where *s*_*j *_denote the index for representing *k *selected genes for *j *= 1,...,*k*. The selected similar genes have complete values without missing observations. Then,

(g*Tgs1Tgs2T⋮gskT)=(αy1y2⋯yN−1ws1xs1,1xs1,2⋯xs1,N−1⋮⋮⋮⋮⋮wskxsk,1xsk,2⋯xsk,N−1)=(αyTwX)     (1)
 MathType@MTEF@5@5@+=feaafiart1ev1aaatCvAUfKttLearuWrP9MDH5MBPbIqV92AaeXatLxBI9gBaebbnrfifHhDYfgasaacH8akY=wiFfYdH8Gipec8Eeeu0xXdbba9frFj0=OqFfea0dXdd9vqai=hGuQ8kuc9pgc9s8qqaq=dirpe0xb9q8qiLsFr0=vr0=vr0dc8meaabaqaciaacaGaaeqabaqabeGadaaakeaadaqadaqaauaabeqafeaaaaqaaiabhEgaNnaaCaaaleqabaGaeCOkaOIaemivaqfaaaGcbaGaeC4zaC2aa0baaSqaaiabhohaZnaaBaaameaacqWHXaqmaeqaaaWcbaGaemivaqfaaaGcbaGaeC4zaC2aa0baaSqaaiabhohaZnaaBaaameaacqWHYaGmaeqaaaWcbaGaemivaqfaaaGcbaGaeSO7I0eabaGaeC4zaC2aa0baaSqaaiabhohaZnaaBaaameaacqWHRbWAaeqaaaWcbaGaemivaqfaaaaaaOGaayjkaiaawMcaaiabg2da9maabmaabaqbaeqabqqbaaaaaeaaiiGacqWFXoqyaeaacqWG5bqEdaWgaaWcbaGaeGymaedabeaaaOqaaiabdMha5naaBaaaleaacqaIYaGmaeqaaaGcbaGaeS47IWeabaGaemyEaK3aaSbaaSqaaiabd6eaojabgkHiTiabigdaXaqabaaakeaacqWG3bWDdaWgaaWcbaGaem4Cam3aaSbaaWqaaiabigdaXaqabaaaleqaaaGcbaGaemiEaG3aaSbaaSqaaiabdohaZnaaBaaameaacqaIXaqmaeqaaSGaeiilaWIaeGymaedabeaaaOqaaiabdIha4naaBaaaleaacqWGZbWCdaWgaaadbaGaeGymaedabeaaliabcYcaSiabikdaYaqabaaakeaacqWIVlctaeaacqWG4baEdaWgaaWcbaGaem4Cam3aaSbaaWqaaiabigdaXaqabaWccqGGSaalcqWGobGtcqGHsislcqaIXaqmaeqaaaGcbaGaeSO7I0eabaGaeSO7I0eabaGaeSO7I0eabaGaeSO7I0eabaGaeSO7I0eabaGaem4DaC3aaSbaaSqaaiabdohaZnaaBaaameaacqWGRbWAaeqaaaWcbeaaaOqaaiabdIha4naaBaaaleaacqWGZbWCdaWgaaadbaGaem4AaSgabeaaliabcYcaSiabigdaXaqabaaakeaacqWG4baEdaWgaaWcbaGaem4Cam3aaSbaaWqaaiabdUgaRbqabaWccqGGSaalcqaIYaGmaeqaaaGcbaGaeS47IWeabaGaemiEaG3aaSbaaSqaaiabdohaZnaaBaaameaacqWGRbWAaeqaaSGaeiilaWIaemOta4KaeyOeI0IaeGymaedabeaaaaaakiaawIcacaGLPaaacqGH9aqpdaqadaqaauaabeqaciaaaeaacqWFXoqyaeaacqWH5bqEdaahaaWcbeqaaiabdsfaubaaaOqaaiabhEha3bqaaiabhIfaybaaaiaawIcacaGLPaaacaWLjaGaaCzcamaabmaabaGaeGymaedacaGLOaGaayzkaaaaaa@A2AD@

where w=[ws1,ws2,⋯,wsk]T
 MathType@MTEF@5@5@+=feaafiart1ev1aaatCvAUfKttLearuWrP9MDH5MBPbIqV92AaeXatLxBI9gBaebbnrfifHhDYfgasaacH8akY=wiFfYdH8Gipec8Eeeu0xXdbba9frFj0=OqFfea0dXdd9vqai=hGuQ8kuc9pgc9s8qqaq=dirpe0xb9q8qiLsFr0=vr0=vr0dc8meaabaqaciaacaGaaeqabaqabeGadaaakeaacqWH3bWDcqGH9aqpcqGGBbWwcqWG3bWDdaWgaaWcbaGaem4Cam3aaSbaaWqaaiabigdaXaqabaaaleqaaOGaeiilaWIaem4DaC3aaSbaaSqaaiabdohaZnaaBaaameaacqaIYaGmaeqaaaWcbeaakiabcYcaSiabl+UimjabcYcaSiabdEha3naaBaaaleaacqWGZbWCdaWgaaadbaGaem4AaSgabeaaaSqabaGccqGGDbqxdaahaaWcbeqaaiabdsfaubaaaaa@44D6@, and **y **= [*y*_1_, *y*_2_,...,*y*_*N*-1_]^*T *^is a subvector of g* excluding the missing value *α*.

LLSimpute selects the *k *most similar genes using *L*_2_-norm or Pearson correlation and applies multiple regression to impute missing values with a linear combination of the *k *selected genes. LLSimpute applies multiple regression in two ways.

The first model is

**y **= **X**^*T*^*β *+ *ε*

α^=wTβ^=wT(XXT)−Xy     (2)
 MathType@MTEF@5@5@+=feaafiart1ev1aaatCvAUfKttLearuWrP9MDH5MBPbIqV92AaeXatLxBI9gBaebbnrfifHhDYfgasaacH8akY=wiFfYdH8Gipec8Eeeu0xXdbba9frFj0=OqFfea0dXdd9vqai=hGuQ8kuc9pgc9s8qqaq=dirpe0xb9q8qiLsFr0=vr0=vr0dc8meaabaqaciaacaGaaeqabaqabeGadaaakeaaiiGacuWFXoqygaqcaiabg2da9iabhEha3naaCaaaleqabaGaemivaqfaaOGaf8NSdiMbaKaacqGH9aqpcqWH3bWDdaahaaWcbeqaaiabdsfaubaakiabcIcaOiabhIfayjabhIfaynaaCaaaleqabaGaemivaqfaaOGaeiykaKYaaWbaaSqabeaacqGHsislaaGccqWHybawcqWH5bqEcaWLjaGaaCzcamaabmaabaGaeGOmaidacaGLOaGaayzkaaaaaa@4513@

and the second model is

**w **= **X**β* + ε*

α^*=yTβ^*=yT(XTX)−XTw     (3)
 MathType@MTEF@5@5@+=feaafiart1ev1aaatCvAUfKttLearuWrP9MDH5MBPbIqV92AaeXatLxBI9gBaebbnrfifHhDYfgasaacH8akY=wiFfYdH8Gipec8Eeeu0xXdbba9frFj0=OqFfea0dXdd9vqai=hGuQ8kuc9pgc9s8qqaq=dirpe0xb9q8qiLsFr0=vr0=vr0dc8meaabaqaciaacaGaaeqabaqabeGadaaakeaaiiGacuWFXoqygaqcamaaCaaaleqabaGaeiOkaOcaaOGaeyypa0JaeCyEaK3aaWbaaSqabeaacqWGubavaaGccuWFYoGygaqcamaaCaaaleqabaGaeiOkaOcaaOGaeyypa0JaeCyEaK3aaWbaaSqabeaacqWGubavaaGccqGGOaakcqWHybawdaahaaWcbeqaaiabdsfaubaakiabhIfayjabcMcaPmaaCaaaleqabaGaeyOeI0caaOGaeCiwaG1aaWbaaSqabeaacqWGubavaaGccqWH3bWDcaWLjaGaaCzcamaabmaabaGaeG4mamdacaGLOaGaayzkaaaaaa@48A7@

where (**XX**^*T*^)^- ^is the generalized inverse of (**XX**^*T*^). If N is larger than k, (**XX**^*T*^)^- ^in the first model is easier to calculate (**X**^*T*^**X**)^- ^than in the second model and vice versa.

In case of multiple missing values in a gene, all missing components of each gene are excluded to find similar genes. Then, the vectors **w **and **y**^*T*^, and matrix **X **are formed in a similar way as in the case of one missing entry, only with different dimensions.

LLSimpute showed a good performance for a relatively large value of *k*. However, if a value of *k *is close to the number of samples, LLSimpute performed poorly compared to other imputation methods. It is probably due to the multi-collinearity problem that LLSimpute performs poorly when *k *is small. The patterns of gene expression are highly correlated leading to the poor performance of multiple regression.

To overcome this limitation, we perform a regression with the principal components rather than the original data. Our technique utilizes the selection of two models in terms of *k *and applies the principal components analysis to the *k *selected genes. Also we consider the robustness to reduce the effects of the outliers by fitting robust regression.

The RLSP method consists of three parts: (1) selection of *k *similar genes, (2) principal component analysis with the *k *selected genes, and (3) robust regression analysis using these principal components. We describe these processes step by step.

### STEP 1 : Selection of *k *similar genes

To impute a missing value *α*, the *k *similar genes are used for RLSP, where *k *is a pre-determined number. In LLSimpute, *L*_2_-norm or Pearson correlation coefficient is used to select *k *similar genes. However, it is well known that *L*_2_-norm and Pearson correlation coefficients are sensitive to outliers. In RLSP, we use *L*_1_-norm as a distance measure to select the *k *similar genes for imputing the missing values of the gene g*,

d(g*,gi)=∑j=1N−1|xi,j−yj|.
 MathType@MTEF@5@5@+=feaafiart1ev1aaatCvAUfKttLearuWrP9MDH5MBPbIqV92AaeXatLxBI9gBaebbnrfifHhDYfgasaacH8akY=wiFfYdH8Gipec8Eeeu0xXdbba9frFj0=OqFfea0dXdd9vqai=hGuQ8kuc9pgc9s8qqaq=dirpe0xb9q8qiLsFr0=vr0=vr0dc8meaabaqaciaacaGaaeqabaqabeGadaaakeaacqWGKbazcqGGOaakcqWHNbWzdaahaaWcbeqaaiabcQcaQaaakiabcYcaSiabhEgaNnaaBaaaleaacqWGPbqAaeqaaOGaeiykaKIaeyypa0ZaaabCaeaadaabdaqaaiabdIha4naaBaaaleaacqWGPbqAcqGGSaalcqWGQbGAaeqaaOGaeyOeI0IaemyEaK3aaSbaaSqaaiabdQgaQbqabaaakiaawEa7caGLiWoaaSqaaiabdQgaQjabg2da9iabigdaXaqaaiabd6eaojabgkHiTiabigdaXaqdcqGHris5aOGaeiOla4caaa@4CD8@

### STEP 2 : Principal component

After selecting *k *similar genes, we perform the principal component analysis. We define two types of variance-covariance matrix. The first one is a *k *× *k *matrix

V=∑i=1N−1∑j=1N−1(xi−x¯.)(xj−x¯.)T     (4)
 MathType@MTEF@5@5@+=feaafiart1ev1aaatCvAUfKttLearuWrP9MDH5MBPbIqV92AaeXatLxBI9gBaebbnrfifHhDYfgasaacH8akY=wiFfYdH8Gipec8Eeeu0xXdbba9frFj0=OqFfea0dXdd9vqai=hGuQ8kuc9pgc9s8qqaq=dirpe0xb9q8qiLsFr0=vr0=vr0dc8meaabaqaciaacaGaaeqabaqabeGadaaakeaacqWGwbGvcqGH9aqpdaaeWbqaamaaqahabaGaeiikaGIaeCiEaG3aaSbaaSqaaiabdMgaPbqabaGccqGHsislcuWH4baEgaqeaiabc6caUaWcbaGaemOAaOMaeyypa0JaeGymaedabaGaemOta4KaeyOeI0IaeGymaedaniabggHiLdGccqGGPaqkcqGGOaakcqWH4baEdaWgaaWcbaGaemOAaOgabeaakiabgkHiTiqbhIha4zaaraGaeiOla4IaeiykaKYaaWbaaSqabeaacqWGubavaaGccaWLjaGaaCzcamaabmaabaGaeGinaqdacaGLOaGaayzkaaaaleaacqWGPbqAcqGH9aqpcqaIXaqmaeaacqWGobGtcqGHsislcqaIXaqma0GaeyyeIuoaaaa@5596@

where xi=[xs1,i,xs2,i,⋯,xsk,i]T, x¯.=[x¯s1,.,x¯s2,.,⋯,x¯sk,.]T
 MathType@MTEF@5@5@+=feaafiart1ev1aaatCvAUfKttLearuWrP9MDH5MBPbIqV92AaeXatLxBI9gBaebbnrfifHhDYfgasaacH8akY=wiFfYdH8Gipec8Eeeu0xXdbba9frFj0=OqFfea0dXdd9vqai=hGuQ8kuc9pgc9s8qqaq=dirpe0xb9q8qiLsFr0=vr0=vr0dc8meaabaqaciaacaGaaeqabaqabeGadaaakeaacqWH4baEdaWgaaWcbaGaemyAaKgabeaakiabg2da9iabcUfaBjabdIha4naaBaaaleaacqWGZbWCdaWgaaadbaGaeGymaedabeaaliabcYcaSiabdMgaPbqabaGccqGGSaalcqWG4baEdaWgaaWcbaGaem4Cam3aaSbaaWqaaiabikdaYaqabaWccqGGSaalcqWGPbqAaeqaaOGaeiilaWIaeS47IWKaeiilaWIaemiEaG3aaSbaaSqaaiabdohaZnaaBaaameaacqWGRbWAaeqaaSGaeiilaWIaemyAaKgabeaakiabc2faDnaaCaaaleqabaGaemivaqfaaOGaeiilaWIaeeiiaaIafCiEaGNbaebacqGGUaGlcqGH9aqpcqGGBbWwcuWG4baEgaqeamaaBaaaleaacqWGZbWCdaWgaaadbaGaeGymaedabeaaliabcYcaSiabc6caUaqabaGccqGGSaalcuWG4baEgaqeamaaBaaaleaacqWGZbWCdaWgaaadbaGaeGOmaidabeaaliabcYcaSiabc6caUaqabaGccqGGSaalcqWIVlctcqGGSaalcuWG4baEgaqeamaaBaaaleaacqWGZbWCdaWgaaadbaGaem4AaSgabeaaliabcYcaSiabc6caUaqabaGccqGGDbqxdaahaaWcbeqaaiabdsfaubaaaaa@6D93@, and  x¯sl,.=1N−1∑i=1N−1xsl,i
 MathType@MTEF@5@5@+=feaafiart1ev1aaatCvAUfKttLearuWrP9MDH5MBPbIqV92AaeXatLxBI9gBaebbnrfifHhDYfgasaacH8akY=wiFfYdH8Gipec8Eeeu0xXdbba9frFj0=OqFfea0dXdd9vqai=hGuQ8kuc9pgc9s8qqaq=dirpe0xb9q8qiLsFr0=vr0=vr0dc8meaabaqaciaacaGaaeqabaqabeGadaaakeaacqqGGaaicuWG4baEgaqeamaaBaaaleaacqWGZbWCdaWgaaadbaGaemiBaWgabeaaliabcYcaSiabc6caUaqabaGccqGH9aqpdaWcaaqaaiabigdaXaqaaiabd6eaojabgkHiTiabigdaXaaadaaeWaqaaiabdIha4naaBaaaleaacqWGZbWCdaWgaaadbaGaemiBaWgabeaaliabcYcaSiabdMgaPbqabaaabaGaemyAaKMaeyypa0JaeGymaedabaGaemOta4KaeyOeI0IaeGymaedaniabggHiLdaaaa@4840@. The second type is a (*N*-1) × (*N*-1) matrix

V*=∑i=1k∑j=1k(xi*−x¯.*)(xj*−x¯.*)T     (5)
 MathType@MTEF@5@5@+=feaafiart1ev1aaatCvAUfKttLearuWrP9MDH5MBPbIqV92AaeXatLxBI9gBaebbnrfifHhDYfgasaacH8akY=wiFfYdH8Gipec8Eeeu0xXdbba9frFj0=OqFfea0dXdd9vqai=hGuQ8kuc9pgc9s8qqaq=dirpe0xb9q8qiLsFr0=vr0=vr0dc8meaabaqaciaacaGaaeqabaqabeGadaaakeaacqWGwbGvdaahaaWcbeqaaiabcQcaQaaakiabg2da9maaqahabaWaaabCaeaacqGGOaakcqWH4baEdaqhaaWcbaGaemyAaKgabaGaeiOkaOcaaOGaeyOeI0IafCiEaGNbaebadaqhaaWcbaGaeiOla4cabaGaeiOkaOcaaaqaaiabdQgaQjabg2da9iabigdaXaqaaiabdUgaRbqdcqGHris5aOGaeiykaKIaeiikaGIaeCiEaG3aa0baaSqaaiabdQgaQbqaaiabcQcaQaaakiabgkHiTiqbhIha4zaaraWaa0baaSqaaiabc6caUaqaaiabcQcaQaaakiabcMcaPmaaCaaaleqabaGaemivaqfaaOGaaCzcaiaaxMaadaqadaqaaiabiwda1aGaayjkaiaawMcaaaWcbaGaemyAaKMaeyypa0JaeGymaedabaGaem4AaSganiabggHiLdaaaa@5730@

where xi*=[xsi,1,xsi,2,⋯,xsi,N−1]T, x¯.*=[x¯.,1,x¯.,2,⋯,x¯.,N−1]T
 MathType@MTEF@5@5@+=feaafiart1ev1aaatCvAUfKttLearuWrP9MDH5MBPbIqV92AaeXatLxBI9gBamXvP5wqSXMqHnxAJn0BKvguHDwzZbqegyvzYrwyUfgarqqtubsr4rNCHbGeaGqiA8vkIkVAFgIELiFeLkFeLk=iY=Hhbbf9v8qqaqFr0xc9pk0xbba9q8WqFfeaY=biLkVcLq=JHqVepeea0=as0db9vqpepesP0xe9Fve9Fve9GapdbaqaaeGacaGaaiaabeqaamqadiabaaGcbaGaeCiEaG3aa0baaSqaaiabdMgaPbqaaiabcQcaQaaakiabg2da9iabcUfaBjabdIha4naaBaaaleaacqWGZbWCdaWgaaadbaGaemyAaKgabeaaliabcYcaSiabigdaXaqabaGccqGGSaalcqWG4baEdaWgaaWcbaGaem4Cam3aaSbaaWqaaiabdMgaPbqabaWccqGGSaalcqaIYaGmaeqaaOGaeiilaWIaeS47IWKaeiilaWIaemiEaG3aaSbaaSqaaiabdohaZnaaBaaameaacqWGPbqAaeqaaSGaeiilaWIaemOta4KaeyOeI0IaeGymaedabeaakiabc2faDnaaCaaaleqabaGaemivaqfaaOGaeiilaWIaeeiiaaIafCiEaGNbaebadaqhaaWcbaGaeiOla4cabaGaeiOkaOcaaOGaeyypa0Jaei4waSLafmiEaGNbaebadaWgaaWcbaGaeiOla4IaeiilaWIaeGymaedabeaakiabcYcaSiqbdIha4zaaraWaaSbaaSqaaiabc6caUiabcYcaSiabikdaYaqabaGccqGGSaalcqWIVlctcqGGSaalcuWG4baEgaqeamaaBaaaleaacqGGUaGlcqGGSaalcqWGobGtcqGHsislcqaIXaqmaeqaaOGaeiyxa01aaWbaaSqabeaacqWGubavaaaaaa@7E13@, and x¯.,l=1k∑i=1kxsi,l
 MathType@MTEF@5@5@+=feaafiart1ev1aaatCvAUfKttLearuWrP9MDH5MBPbIqV92AaeXatLxBI9gBaebbnrfifHhDYfgasaacH8akY=wiFfYdH8Gipec8Eeeu0xXdbba9frFj0=OqFfea0dXdd9vqai=hGuQ8kuc9pgc9s8qqaq=dirpe0xb9q8qiLsFr0=vr0=vr0dc8meaabaqaciaacaGaaeqabaqabeGadaaakeaacuWG4baEgaqeamaaBaaaleaacqGGUaGlcqGGSaalcqWGSbaBaeqaaOGaeyypa0ZaaSaaaeaacqaIXaqmaeaacqWGRbWAaaWaaabmaeaacqWG4baEdaWgaaWcbaGaem4Cam3aaSbaaWqaaiabdMgaPbqabaWccqGGSaalcqWGSbaBaeqaaaqaaiabdMgaPjabg2da9iabigdaXaqaaiabdUgaRbqdcqGHris5aaaa@428C@.

When *k *is larger than *N*, the size of *V *matrix becomes too large to handle and it is not computationally efficient to derive the principal components. Therefore, we use V* instead of *V *and use a different type of regression. *V *corresponds to the first model and V* corresponds to the second model in LLS impute, respectively. Kim et al. [4] showed that the solutions based on V and V* are in fact the same. Let **PC**_*x *_= {*PC*_1_, *PC*_2_,...,*PC*_*k*_} be the principal components using *V *and PCx*={PC1*,PC2*,⋯,PCN−1*}
 MathType@MTEF@5@5@+=feaafiart1ev1aaatCvAUfKttLearuWrP9MDH5MBPbIqV92AaeXatLxBI9gBaebbnrfifHhDYfgasaacH8akY=wiFfYdH8Gipec8Eeeu0xXdbba9frFj0=OqFfea0dXdd9vqai=hGuQ8kuc9pgc9s8qqaq=dirpe0xb9q8qiLsFr0=vr0=vr0dc8meaabaqaciaacaGaaeqabaqabeGadaaakeaacqWHqbaucqWHdbWqdaqhaaWcbaGaemiEaGhabaGaeiOkaOcaaOGaeyypa0Jaei4EaSNaemiuaaLaem4qam0aa0baaSqaaiabigdaXaqaaiabcQcaQaaakiabcYcaSiabdcfaqjabdoeadnaaDaaaleaacqaIYaGmaeaacqGGQaGkaaGccqGGSaalcqWIVlctcqGGSaalcqWGqbaucqWGdbWqdaqhaaWcbaGaemOta4KaeyOeI0IaeGymaedabaGaeiOkaOcaaOGaeiyFa0haaa@48D1@ be the principal components using V*. Then, these principal components **PC**_*x *_and PCx*
 MathType@MTEF@5@5@+=feaafiart1ev1aaatCvAUfKttLearuWrP9MDH5MBPbIqV92AaeXatLxBI9gBaebbnrfifHhDYfgasaacH8akY=wiFfYdH8Gipec8Eeeu0xXdbba9frFj0=OqFfea0dXdd9vqai=hGuQ8kuc9pgc9s8qqaq=dirpe0xb9q8qiLsFr0=vr0=vr0dc8meaabaqaciaacaGaaeqabaqabeGadaaakeaacqWHqbaucqWHdbWqdaqhaaWcbaGaemiEaGhabaGaeiOkaOcaaaaa@316E@ are used for the first type of regression (equation (2)) and the second type of regression model (equation (3)), respectively.

### STEP 3 : Robust regression

We use *PC*_1_, *PC*_2_,...,*PC*_*p *_or PC1*,PC2*,⋯,PCp*
 MathType@MTEF@5@5@+=feaafiart1ev1aaatCvAUfKttLearuWrP9MDH5MBPbIqV92AaeXatLxBI9gBaebbnrfifHhDYfgasaacH8akY=wiFfYdH8Gipec8Eeeu0xXdbba9frFj0=OqFfea0dXdd9vqai=hGuQ8kuc9pgc9s8qqaq=dirpe0xb9q8qiLsFr0=vr0=vr0dc8meaabaqaciaacaGaaeqabaqabeGadaaakeaacqWGqbaucqWGdbWqdaqhaaWcbaGaeGymaedabaGaeiOkaOcaaOGaeiilaWIaemiuaaLaem4qam0aa0baaSqaaiabikdaYaqaaiabcQcaQaaakiabcYcaSiabl+UimjabcYcaSiabdcfaqjabdoeadnaaDaaaleaacqWGWbaCaeaacqGGQaGkaaaaaa@3E5C@ as new exploratory variables and fit the regression model in a robust manner, where *p *is the predetermined number of the principal components. The corresponding regression models are

y=∑i=1pPCiβi+ε     (6)
 MathType@MTEF@5@5@+=feaafiart1ev1aaatCvAUfKttLearuWrP9MDH5MBPbIqV92AaeXatLxBI9gBaebbnrfifHhDYfgasaacH8akY=wiFfYdH8Gipec8Eeeu0xXdbba9frFj0=OqFfea0dXdd9vqai=hGuQ8kuc9pgc9s8qqaq=dirpe0xb9q8qiLsFr0=vr0=vr0dc8meaabaqaciaacaGaaeqabaqabeGadaaakeaacqWH5bqEcqGH9aqpdaaeWbqaaiabdcfaqjabdoeadnaaBaaaleaacqWGPbqAaeqaaGGacOGae8NSdi2aaSbaaSqaaiabdMgaPbqabaaabaGaemyAaKMaeyypa0JaeGymaedabaGaemiCaahaniabggHiLdGccqGHRaWkcqWF1oqzcaWLjaGaaCzcamaabmaabaGaeGOnaydacaGLOaGaayzkaaaaaa@436F@

and

w=∑i=1pPCi*βi*+ε*     (7)
 MathType@MTEF@5@5@+=feaafiart1ev1aaatCvAUfKttLearuWrP9MDH5MBPbIqV92AaeXatLxBI9gBamXvP5wqSXMqHnxAJn0BKvguHDwzZbqegyvzYrwyUfgarqqtubsr4rNCHbGeaGqiA8vkIkVAFgIELiFeLkFeLk=iY=Hhbbf9v8qqaqFr0xc9pk0xbba9q8WqFfeaY=biLkVcLq=JHqVepeea0=as0db9vqpepesP0xe9Fve9Fve9GapdbaqaaeGacaGaaiaabeqaamqadiabaaGcbaGaeC4DaCNaeyypa0ZaaabCaeaacqWGqbaucqWGdbWqdaqhaaWcbaGaemyAaKgabaGaeiOkaOcaaGGacOGae8NSdi2aa0baaSqaaiabdMgaPbqaaiabcQcaQaaaaeaacqWGPbqAcqGH9aqpcqaIXaqmaeaacqWGWbaCa0GaeyyeIuoakiabgUcaRiab=v7aLnaaCaaaleqabaGaeiOkaOcaaOGaaCzcaiaaxMaadaqadaqaaiabiEda3aGaayjkaiaawMcaaaaa@5679@

In our method, we use a quantile regression to fit the regression model in a robust manner. Robust regression usually provides an alternative analysis to least square regression when fundamental assumptions such as normality or variance homogeneity are violated. The quantile regression using the 50th percentile estimates the model parameters by minimizing the sum of absolute values of the residuals [10]. It is estimated by minimizing ∑j=1N−1|yj−∑i=1pPCijβi|
 MathType@MTEF@5@5@+=feaafiart1ev1aaatCvAUfKttLearuWrP9MDH5MBPbIqV92AaeXatLxBI9gBaebbnrfifHhDYfgasaacH8akY=wiFfYdH8Gipec8Eeeu0xXdbba9frFj0=OqFfea0dXdd9vqai=hGuQ8kuc9pgc9s8qqaq=dirpe0xb9q8qiLsFr0=vr0=vr0dc8meaabaqaciaacaGaaeqabaqabeGadaaakeaadaaeWaqaamaaemaabaGaemyEaK3aaSbaaSqaaiabdQgaQbqabaGccqGHsisldaaeWaqaaiabdcfaqjabdoeadnaaBaaaleaacqWGPbqAcqWGQbGAaeqaaGGacOGae8NSdi2aaSbaaSqaaiabdMgaPbqabaaabaGaemyAaKMaeyypa0JaeGymaedabaGaemiCaahaniabggHiLdaakiaawEa7caGLiWoaaSqaaiabdQgaQjabg2da9iabigdaXaqaaiabd6eaojabgkHiTiabigdaXaqdcqGHris5aaaa@4B30@ or ∑j=1k|wsj−∑i=1pPCij*βi*|
 MathType@MTEF@5@5@+=feaafiart1ev1aaatCvAUfKttLearuWrP9MDH5MBPbIqV92AaeXatLxBI9gBaebbnrfifHhDYfgasaacH8akY=wiFfYdH8Gipec8Eeeu0xXdbba9frFj0=OqFfea0dXdd9vqai=hGuQ8kuc9pgc9s8qqaq=dirpe0xb9q8qiLsFr0=vr0=vr0dc8meaabaqaciaacaGaaeqabaqabeGadaaakeaadaaeWaqaamaaemaabaGaem4DaC3aaSbaaSqaaiabdohaZnaaBaaameaacqWGQbGAaeqaaaWcbeaakiabgkHiTmaaqadabaGaemiuaaLaem4qam0aa0baaSqaaiabdMgaPjabdQgaQbqaaiabcQcaQaaaiiGakiab=j7aInaaDaaaleaacqWGPbqAaeaacqGGQaGkaaaabaGaemyAaKMaeyypa0JaeGymaedabaGaemiCaahaniabggHiLdaakiaawEa7caGLiWoaaSqaaiabdQgaQjabg2da9iabigdaXaqaaiabdUgaRbqdcqGHris5aaaa@4CEA@. This way our analysis method can reject outliers and maintains robustness.

If *p *= *k *and the regression model is estimated by the least squares method, RLSP is the same as LLSimpute. When *k *is small, we recommend using *p *= 1 and fit the regression model *y*_*i *_= *β*_1_*PC*_1*i *_+ *ε*_*i *_using the sum of least absolute deviations. The imputed value of *α *is defined by α^=β^1PCw
 MathType@MTEF@5@5@+=feaafiart1ev1aaatCvAUfKttLearuWrP9MDH5MBPbIqV92AaeXatLxBI9gBaebbnrfifHhDYfgasaacH8akY=wiFfYdH8Gipec8Eeeu0xXdbba9frFj0=OqFfea0dXdd9vqai=hGuQ8kuc9pgc9s8qqaq=dirpe0xb9q8qiLsFr0=vr0=vr0dc8meaabaqaciaacaGaaeqabaqabeGadaaakeaaiiGacuWFXoqygaqcaiabg2da9iqb=j7aIzaajaWaaSbaaSqaaiabigdaXaqabaGccqWGqbaucqWGdbWqdaWgaaWcbaGaem4DaChabeaaaaa@3615@ where *PC*_*w *_is the projected data of **w **onto the direction of *PC*_1_. For *k *much larger than *N*, we recommend using *p *close to the number of the sample size.

## Results and discussion

### Datasets

Four data sets are used for the comparative study: three Spellman data sets (ALPHA, ELU, and ALPHA+ELU, [5,11]), and Gasch data set [12]. These data sets were also used in the comparative study of LLSimpute [4]. ALPHA dataset was obtained from *α*-factor block release studied for the identification of cell-cycle regulated genes in yeast *Saccharomyces cerevisiae *[13]. ELU dataset was elutriation dataset in the same study. After removing all the genes with missing values in the ALPHA and ELU datasets, we obtained complete data matrices that contain 4,304 genes and 18 experiments and 4,304 genes and 14 experiments, respectively. ALPHA+ELU dataset was used for the examination of the additional sample effects as studied Oba et al. [5]. Gasch dataset was obtained from the study of genomic expression responses to DNA damage [14]. After removing all genes with missing values, a complete data matrix with 2641 genes and 44 experiments was prepared for this study. For the simulation study, 1% and 5% missing observations were randomly generated in these data sets.

We use the normalized root mean squares error (NRMSE) to evaluate the performances of the missing value imputation approaches, computed by

NRMSE =mean[(yguess−yanswer)2]/sd(yanswer),
 MathType@MTEF@5@5@+=feaafiart1ev1aaatCvAUfKttLearuWrP9MDH5MBPbIqV92AaeXatLxBI9gBaebbnrfifHhDYfgasaacH8akY=wiFfYdH8Gipec8Eeeu0xXdbba9frFj0=OqFfea0dXdd9vqai=hGuQ8kuc9pgc9s8qqaq=dirpe0xb9q8qiLsFr0=vr0=vr0dc8meaabaqaciaacaGaaeqabaqabeGadaaakeaacqqGobGtcqqGsbGucqqGnbqtcqqGtbWucqqGfbqrcqqGGaaicqqG9aqpdaWcgaqaamaakaaabaGaemyBa0MaemyzauMaemyyaeMaemOBa4Maei4waSLaeiikaGIaemyEaK3aaSbaaSqaaiabbEgaNjabbwha1jabbwgaLjabbohaZjabbohaZbqabaGccqGHsislcqWG5bqEdaWgaaWcbaGaeeyyaeMaeeOBa4Maee4CamNaee4DaCNaeeyzauMaeeOCaihabeaakiabcMcaPmaaCaaaleqabaGaeGOmaidaaOGaeiyxa0faleqaaaGcbaGaem4CamNaemizaqMaeiikaGIaemyEaK3aaSbaaSqaaiabbggaHjabb6gaUjabbohaZjabbEha3jabbwgaLjabbkhaYbqabaGccqGGPaqkaaGaeiilaWcaaa@61F4@

where *y*_*guess *_is the imputed value and *y*_*answer *_the true value.

### Experimental results

**Figure 1:**
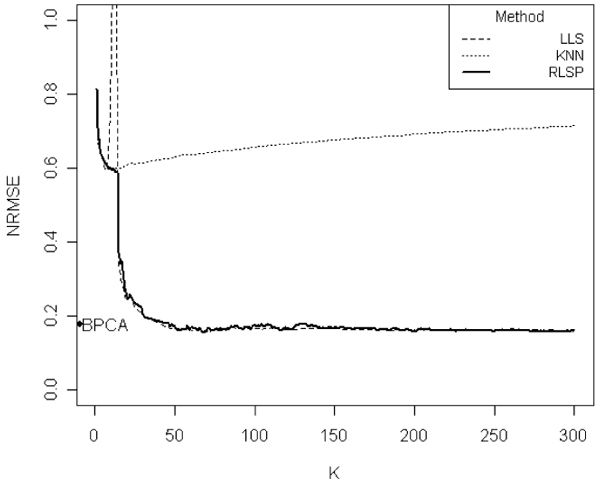
**Comparison of the NRMSEs of various method**s. Comparison of the NRMSEs of LLSimpute, kNN, BPCA, and RLSP imputation methods on ELU data set with the 1% missing rate. BPCA results are shown on the y-axis. The x-axis represents the value of *k *(selected similar genes). When *k *is close to sample size (*N*), LLSimpute has a high peak. Thus we truncated it in the graph. Overall, RLSP method showed improved performance compared to other methods.

**Figure 2:**
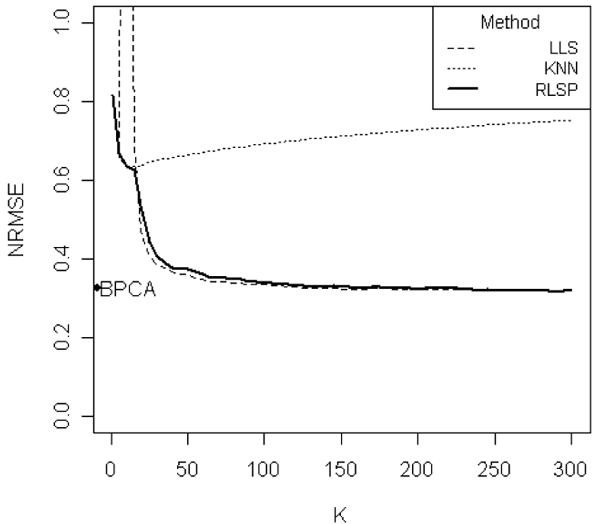
**Comparison of the NRMSEs of various method**s. Comparison of the NRMSEs of LLSimpute, kNN, BPCA, and RLSP imputation methods on ELU data set with the 5% missing rate. BPCA results are shown on the y-axis. The x-axis represents the value of *k *(selected similar genes). When *k *is close to sample size (*N*), LLSimpute has a high peak. Thus we truncated it in the graph. RLSP method demonstrated a better result than other methods.

Figures 1 and 2 show the plots of NRMSE vs. *k *for the ELU Spellman data set, when the missing rates are 1% and 5%, respectively. We compare our RLSP with kNNimpute, LLSimpute and BPCA. For a large *k*, both LLSimpute and RLSP show highly competitive results and perform best compared to kNNimpute and BPCA. However, for a smaller *k*, RLSP performed much better than LLSimpute. LLSimpute shows a high peak when *k *is close to the number of samples. Because highly correlated *k *genes are usually selected in LLSimpute, the poor performance of LLSimpute is probably due to multi-collinearity of the selected *k *genes.

**Figure 3:**
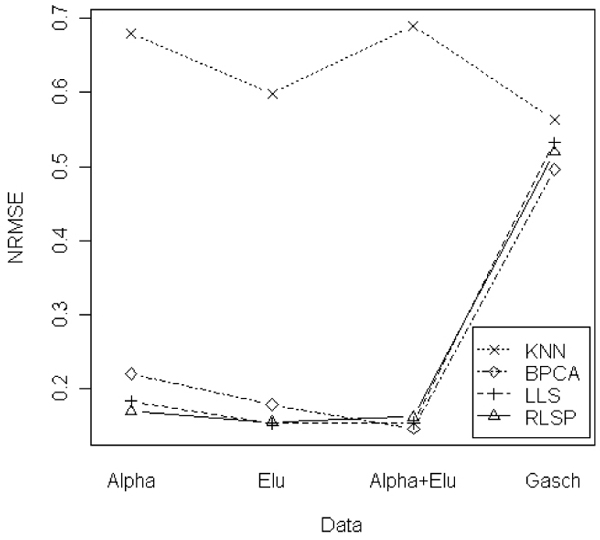
**Comparison of the NRMSEs for 4 different data set**s. Comparison of the NRMSEs for 4 different data sets (ALPHA, ELU, ALPHA+ELU, and Gasch) with 1% missing rate.

**Figure 4:**
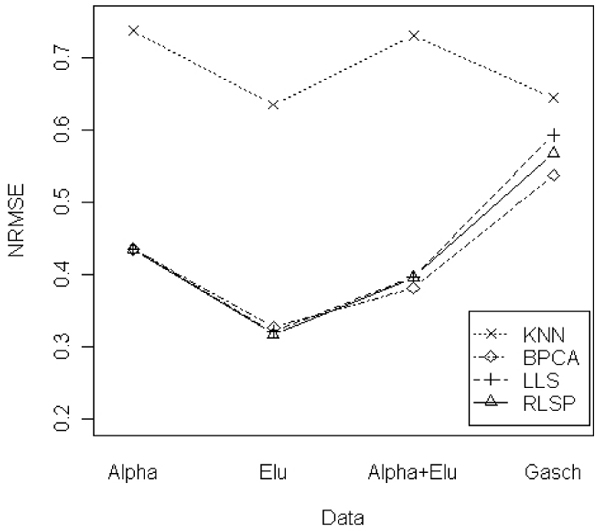
**Comparison of the NRMSEs for 4 different data sets**. Comparison of the NRMSEs for 4 different data sets (ALPHA, ELU, ALPHA+ELU, and Gasch) with 5% missing rate.

Figures 3 and 4 show the smallest NRMSE values for four data sets. Figure 3 represents the results of the case of 1% missing rate. LLSimpute and RLSP showed the similar results and the best performances in ALPHA and ELU data sets. For ALPHA+ELU and Gasch data, BPCA showed a little bit better performance than RLSP and LLSimpute, but it is competitive to LLSimpute and RLSP. Figure 4 shows the results of the case of the 5% missing rate. RLSP, LLSimpute and BPCA show competitive performances for ALPHA and ELU data sets. For large datasets such as ALPHA+ELU and Gasch data sets, BPCA showed a little bit better performance. kNNimpute showed the worst performance in all data sets.

We presume that the differences in the performance of missing value imputation methods are highly dependent on the data set as well as the value of *k*. When a moderate value of *k *is selected, say when *k *is close to the number of samples, the proposed RLSP method outperforms the LLSimpute method on all data sets (data not shown). As the missing rate increases, NRMSEs increase rapidly for all methods and the performances of all four methods become worse.

## Conclusion

The proposed RLSP method was motivated by a similar idea to that of the LLSimpute method. Both methods use the information from the selected *k *genes to estimate missing observations. LLSimpute uses the least squares method using the selected *k *genes. On the other hand, RLSP uses the principal components of the selected genes instead of the original *k *genes, and employs the quantile regression model for a robust analysis. The use of the principal components leads to a large difference between the two methods. The performance of LLSimpute is poor when *k *is small (near the number of samples). Assuming that multi-collinearity is probably the main cause for the poor performance of LLSimpute, RLSP addresses this problem using the principal components and then applying the robust regression approach to reduce the effect of outliers. In summary, RLSP showed more stable performance than LLSimpute for all data sets in our comparative studies.

The performance of RLSP may depend on the value of *p*, the number of principal components. By varying the value of *p*, we examined its effect on the parameter estimators. The result showed that the performance of RLSP is optimal when the value of *p *is close to the number of the sample size (data not shown). A similar result was obtained from a previous study of the BPCA method [5]. However, since the imputation procedure is executed for each missing value of a gene, the optimal value of *p *may differ from gene to gene. Selecting an optimal value for each missing value requires intensive computation. Thus, we recommend using the number of sample size as the value of *p *for practical application, although we expect that an appropriate choice of *p *would improve the performance of the RLSP method.

In terms of computational efficiency, although RLSP and BPCA showed competitive results, BPCA required a higher computational demand due to the EM-like repetitive algorithm. In addition, RLSP seemed less computationally intensive than CMVE. However, a further study based on the same platform would be desirable for the systematic comparison.

The presented method consists of three separate steps, where the first step applied L1 metric to select similar genes, the second step performs PCA on the selected set, which is a L2 method, and finally in the third step L1 metric is applied again to perform robust regression. Among the several combinations of metrics, the proposed combination provided the minimum NRMSE and provided the most computationally efficient result.

The main motivation of the robust regression was to reduce the effect of outliers in estimation of missing observations. Our empirical studies demonstrated that the effect of outliers were not large enough to cause huge differences between robust regression and ordinary regression. Among the several robust regression methods including Tukey's bi-weight M-estimator, the proposed quantile regression using the 50th percentile provided the best result. However, a further study on selecting the better robust method is desirable.

Finally, most missing imputation methods for microarray data assume the simple missing data mechanism to be the so called 'missing completely at random' [15]. However, this mechanism may assume too much to be expected to hold in real applications. Therefore, more complicated methods are required for handling other possible missing data mechanisms. By incorporating the missing data mechanism or missing patterns in the microarray data, we could improve the performance of the missing imputation method.

## Authors' contributions

All authors contributed to the development of RLSP method and the comparative studies with previously proposed imputation methods.
